# miRSponge: a manually curated database for experimentally supported miRNA sponges and ceRNAs

**DOI:** 10.1093/database/bav098

**Published:** 2015-09-30

**Authors:** Peng Wang, Hui Zhi, Yunpeng Zhang, Yue Liu, Jizhou Zhang, Yue Gao, Maoni Guo, Shangwei Ning, Xia Li

**Affiliations:** College of Bioinformatics Science and Technology, Harbin Medical University, Harbin 150081, China

## Abstract

In this study, we describe miRSponge, a manually curated database, which aims at providing an experimentally supported resource for microRNA (miRNA) sponges. Recent evidence suggests that miRNAs are themselves regulated by competing endogenous RNAs (ceRNAs) or ‘miRNA sponges’ that contain miRNA binding sites. These competitive molecules can sequester miRNAs to prevent them interacting with their natural targets to play critical roles in various biological and pathological processes. It has become increasingly important to develop a high quality database to record and store ceRNA data to support future studies. To this end, we have established the experimentally supported miRSponge database that contains data on 599 miRNA-sponge interactions and 463 ceRNA relationships from 11 species following manual curating from nearly 1200 published articles. Database classes include endogenously generated molecules including coding genes, pseudogenes, long non-coding RNAs and circular RNAs, along with exogenously introduced molecules including viral RNAs and artificial engineered sponges. Approximately 70% of the interactions were identified experimentally in disease states. miRSponge provides a user-friendly interface for convenient browsing, retrieval and downloading of dataset. A submission page is also included to allow researchers to submit newly validated miRNA sponge data.

**Database URL**: http://www.bio-bigdata.net/miRSponge.

## Introduction

MicroRNAs (miRNAs) are a class of small (19–25 nt) non-coding RNAs that regulate their target genes by binding to and targeting mRNAs for degradation or by inhibiting protein translation at the post-transcriptional level (1). miRNAs are involved in many biological processes (2–4), and aberrant expression of miRNAs has been implicated in numerous diseases including cancers (2). Emerging evidence suggests that miRNAs are themselves regulated by other RNA molecules that contain complementary miRNA binding sites, such as mRNAs, pseudogenes, long non-coding RNAs (lncRNA) and circular RNAs (5–7). These miRNA-binding RNAs or ‘miRNA sponges’ are competitive regulators that sequester miRNAs which prevents them interacting with their intended targets (8–10). miRNA sponges are known as competing endogenous RNAs (ceRNAs), and they act dynamically to regulate the expression of each other via competing mechanisms that are important for various physiological and pathological processes.

Classes of miRNA sponges include endogenously generated molecules such as coding mRNAs, pseudogenes, lncRNAs and circular RNAs, as well as exogenously introduced molecules such as viral RNAs and artificial engineered sponges (8). For example, several protein-coding genes were found to share miRNAs with the phosphate and tensin homolog (PTEN) and to further activate the PI3K/AKT pathway (11). In another study, the PTEN pseudogene PTENP1 was found to act as a ceRNA, regulating PTEN levels by providing additional miRNA target sites (12). The lncRNA IPS1, originally discovered in plants, acts as a ceRNA by soaking up miR-399 to indirectly control the PHO2 gene which itself encodes a protein that negatively affects phosphate content in the shoots of Arabidopsis (13). An endogenous circular transcript, referred to as ciRS-7 or CDR1as, acts to sequester miR-7 from its natural targets in human brain tissue (14) leading to reduced midbrain size in zebrafish embryos (15). The HSURs transcripts of herpes virus saimiri (HVS), which causes leukemia and lymphoma by infecting T-cells, carry highly conserved miRNA target sites, and downregulate miR-27 expression in HVS-transformed cells (16). Genetic knockout of miRNAs is potentially challenging as some miRNAs are transcribed from more than a single genomic locus (8). Hence, artificial miRNA sponges are often engineered and delivered by vectors as a way to investigate loss-of-function phenotypes or for therapeutic purposes (8, 17). Gentner *et al*. (18) transplanted engineered sponges of myeloid-specific miRNA miR-223 into lethally irradiated mice, and sponge-transplanted mice underwent a myeloid cell expansion and exhibited inflammatory lung pathology comparable to miR-223 knockout mice. The artificial sponge miravirsen, which can sequester and inhibit the liver-specific miR-122, is currently in clinical trials as a potential treatment for hepatitis C virus (19). At present, there is a lack of comprehensive databases that provide a resource for experimentally verified data on miRNA-sponges and ceRNA interactions in different species. To address this issue, we have developed miRSponge, a manually curated database that provides comprehensive information on miRNA sponges and their interacting miRNAs that will be of benefit for future studies and therapeutic strategies.

## Data collection and database content

**Figure 1.**
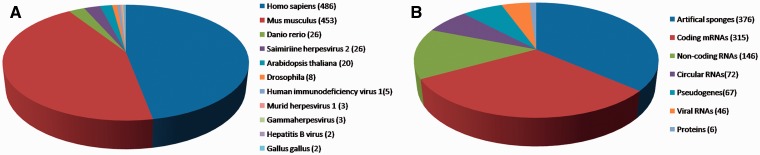
miRSponge statistics. (**A**) A total of 11 species are currently incorporated in miRSponge, including animals, plants and viruses. (**B**) Endogenous and exogenous biological molecules are documented, including coding mRNAs, pseudogenes, lncRNAs, circular RNAs, proteins, viral RNAs and artificial engineered sponges.

We manually collated known miRNA-sponge and ceRNA interactions from the PubMed database using keywords such as ‘miRNA sponges’, ‘miRNA decoys’, ‘antagomirs’ and ‘ceRNA’. Approximately 1200 articles published were extracted and assessed. In the current release of miRSponge (July 2015), 599 miRNA-sponge interactions and 463 ceRNA interactions across 11 species have been documented (Figure 1A). In miRSponge, there are various types of endogenous or exogenous biological molecules documented as miRNA sponges (Figure 1B), including coding mRNAs, pseudogenes, lncRNAs, circular RNAs, proteins, viral RNAs and artificial engineered sponges. Corresponding technical details and how inter-relationships between sponges and miRNAs were collected. High confidence experiments information such as polymerase chain reaction, western blot or luciferase reporter assay, and other reliable methods for the miRSponge records were identified from literature curation.

To maximize information content, a variety of biological annotations were integrated. Each entry includes detailed information on sponges, miRNAs and competing targets, species, miRBase (20) accession, HGNC (21) and NCBI gene (22) annotation, tissue distribution, conditions for detection, associated diseases, experimental methods, corresponding literature, PMIDs and a brief description of the competitive interaction and experimental method used for characterization. miRNA binding sites including sequence alignment score and thermodynamic free energy (predicted by miRanda) on the sponge were provided. Given that some miRNA sponges such as lncRNAs are still poorly understood, functional annotation (Gene Ontology (23) and canonical pathways such as BioCarta (http://www.biocarta.com), KEGG (24), Reactome (25) etc.) including the natural miRNA targets and experimentally validated database entries (TarBase6.0 (26), miRecord (27), miR2Disease (28) and miRTarBase (29)) were included for each sponge. A ‘guilty-by-association’ strategy has been used previously to analyse lncRNA function (30, 31), and miRSponge provides significantly enriched GO terms and pathways for each miRNA sponge (Hypergeometric test, *P* < 0.01). By implementing the DAVID API tool (32), miRSponge allows users to perform multi-context functional analyses. Approximately 70% of the interactions in the database have been confirmed experimentally to be involved in diseases processes. Finally, miRSponge was developed within a J2EE environment, and built using Struts, JSP, tomcat 6.0.33 and MySQL5.0, and runs on a Cent OS 5.5 system.

## User interface

**Figure 2.**
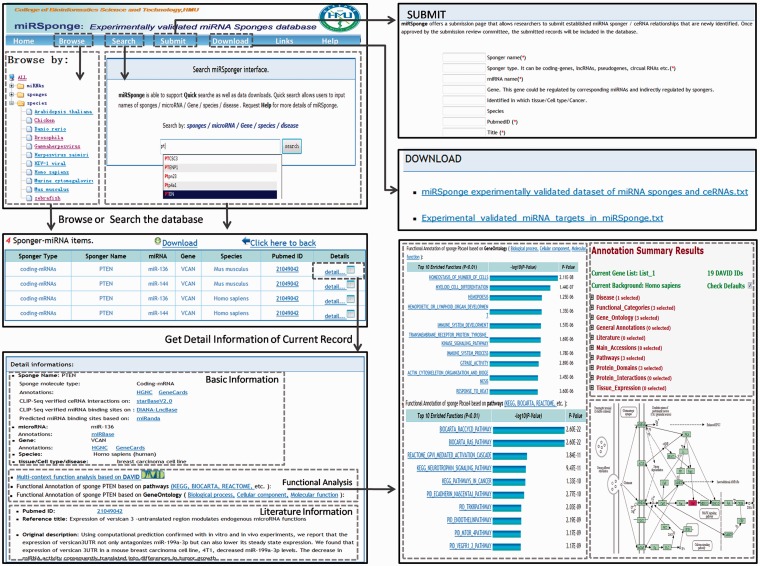
Schematic workflow of the miRSponge database.

The miRSponge database provides a user-friendly interface for database queries (Figure 2). In the search page, users can query a particular miRNA-sponge or ceRNA interaction by inputting optional key words such as sponge name, miRNAs, genes, diseases, tissues, cell lines and species. The search engine supports fuzzy searching, which will list all potential results potentially matching the key words. Further, users can search the database by inputting new RNA sequences in order to identify related miRNA-sponges or ceRNA interactions. To facilitate user analysis, the results of miRNA-sponge and ceRNA relationships are integrated into a non-redundant table, and users can retrieve more extensive information on particularly interesting results by clicking the ‘detail’ button, which leads to a more detailed page containing the corresponding literature, a brief description, visualized functional annotation, among other entries. In addition, miRSponge includes a Browse page for general perusal of the database based on different hierarchical classification miRNA, sponge, species and sponge type trees. By selecting particular nodes, a corresponding results table is displayed, and results tables from all steps can be downloaded at the touch of a ‘Download’ button. The database also provides a ‘Submit’ page that allows users to submit newly identified miRNA-sponge and ceRNA interactions. Once approved by the review committee, new data is included and made available to the public through monthly updates. All associated data sources, including sequence information, experimental validated datasets, functional annotation data, scores and thermodynamic free energy of predicted miRNA targets, can be freely downloaded from the miRSponge ‘Download’ page. The ‘Links’ page provides related resources of miRNA-sponge or ceRNA interactions, such as starBaseV2.0 (31), DIANA-LncBase (33) and LncACTdb (34). A web interface for miRSponge is freely available at http://www.bio-bigdata.net/miRSponge.

## Case studies using the miRSponge database

### An extensive tumor suppressive ceRNA network involving PTEN

**Figure 3.**
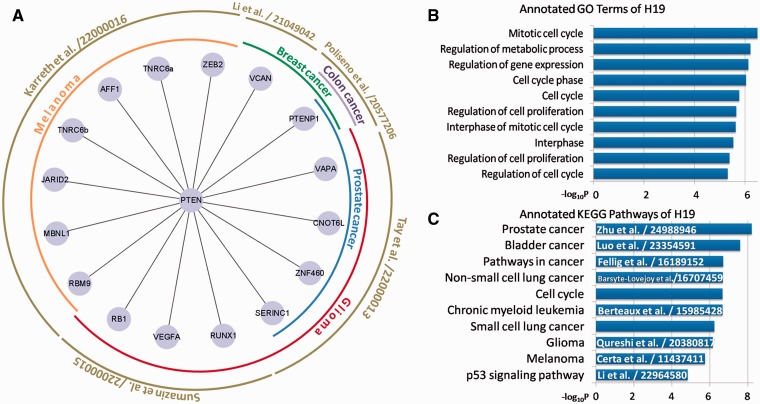
Examples of miRSponge database use. (**A**) PTEN-centered tumor suppressive ceRNA network extracted using miRSponge. (**B**) lncRNA H19 was linked to cell cycle-associated processes and (**C**) Cancer-related pathways, as supported from the existing literature with PubMed ID.

Besides basic searching and downloading from miRSponge, users can perform specific analyses based on miRSponge datasets. For example, the PTEN gene, a critical tumor suppressor that regulates the PI3K/AKT signaling pathway, is regulated by several miRNA sponges in different diseases (12). In this study, we searched the miRSponge database using the key word ‘PTEN’ and used the resulting dataset to construct a PTEN-centered ceRNA network (Figure 3A). In total, 19 sponges were found to be competing with PTEN for miRNAs, according to five separate literature entries. These ceRNA interactions have been validated and shown to regulate different cancers. The miRSponge database provided a more comprehensive source of information on PTEN-associated ceRNAs than could be obtained from the separate articles in the same time. Furthermore, the PTEN-centered ceRNA network indicated a widespread posttranscriptional regulation between ceRNAs that may account for the involvement in tumorigenesis. The PTENP1-PTEN association is involved in various cancers, which indicates a common regulatory activity of the tumor suppression pathway. The extensive regulation of the key tumor suppressor PTEN by ceRNAs may explain why a subtle change in PTEN expression is critical for controlling tumor initiation and progression (11). In summary, this case study showed the fine-tune of the PTEN-centered ceRNA network and to tease out possible contributions to the dysregulation of this complicated biological network that occur during the pathological state.

### Inferring potential functions for miRNA sponges

Based on miRSponge data sources and functional annotation tools, the functions of some poorly understood miRNA sponges can be inferred. LncRNAs fall into this category, and LncRNA H19 was identified as an miRNA sponge containing both canonical and non-canonical binding sites for the let-7 family of miRNAs that play important roles in development, metabolism and cancer (35). By harboring the miRNAs locus, H19 is also a developmental reservoir of miR-675 that suppresses growth and Igf1r (36). To further study the function of H19 and its role in disease, the miRSponge database was searched using the key word ‘H19’ and multi-context functional enrichment analysis was performed using the DAVID API service that is accessible from the database. H19 was found to be involved in cell cycle- (Figure 3B) and cancer-associated pathways (Figure 3C). Dysregulation of the cell cycle can result in aberrant cell growth, proliferation and apoptosis that lead ultimately to cancers. A series of cancer-related pathways including the cell cycle and p53 signaling pathway, and cancer-specific pathways including prostate bladder and lung cancers, were identified (Figure 3B and C). A literature search of the LncRNADisease database (37) found that most of these pathways were previously associated with H19 (Figure 3C), confirming that H19 is an important regulator in many types of cancers and can therefore be used as a potential tumor marker for initial diagnosis and monitoring of therapies. This also further confirmed the usefulness of the miRSponge database.

## Future extensions

miRSponge provides a high quality resource for studying miRNA regulation, and future extensions will further expand the capabilities of this database. Experimentally supported miRNA-sponge/ceRNA association data will be continually added in updates every 2 months, and more species will be included. Additionally, new tools for predicting miRNA-sponge/ceRNA interactions are currently being developed and will be integrated in the future. These tools will identify miRNA-sponge/ceRNA interactions via a bioinformatics pipeline that integrates several *in silico* target predictions, Ago-CLIP experimental data, and expression profiling from transcriptome sequencing experiments.

## Methodology

In miRSponge database, we used miRanda algorithm to find miRNA binding sites. The parameters were set as default (Score ≥140 and Energy ≤ 7.0), which was used in previous studies (38, 39). To provide analysis option of new sequences, miRSponge integrated BLAST method into the searching pipeline. Alignment score of 80% identity was used as threshold to find similar sequence. In functional analysis, ‘guilty-by-association’ strategy was carried out to identify miRNA sponge functions. Eexperimental validated miRNA targets (curated from TarBase6.0 (26), miRecord (27), miR2Disease (28) and miRTarBase (29)) were used for enrichment analysis. We used hypergeometric test to calculate the enrichment significance based on functional context. If the genome context had a total of *N* genes, of which *K* were involved in the function term under investigation, and the set of interesting target genes for analysis had a total of *M* genes, of which *x* were involved in the same function term, then the *P* value can be calculated to evaluate the enrichment significance for that function term as follows:

$$P=1-\sum_{t=0}^{x}\frac{{(}_{t}^{K}){(}_{M-t}^{N-K})}{{(}_{M}^{N})}$$

Significantly enriched functional terms was defined as *P* < 0.05 and further illustrated as bar graph based on −log(P) in the miRSponge detail page. Functional context datasets were download from MSigDB (40).

## Discussion and Conclusions

Increasing numbers of studies have shown that regulatory miRNAs are themselves regulated by other biological molecules (miRNA sponges). These competitive sponges bind to and sequester miRNAs, preventing them from interacting with their natural targets. miRNA sponges are have potential for use in disease diagnosis, prognosis and treatment.

Several works mining interactions between miRNAs and other molecules have been published, such as starBaseV2.0 (31), DIANA-LncBase (33) and LncACTdb (34). These databases focus on interactions between miRNAs and lncRNAs, and identified interactions through a bioinformatics pipeline that integrating data from several *in silico* target prediction studies, Argonaute-CLIP datasets, and expression profiles. The species are restricted to human and mouse. In this work, both endogenous and exogenous biological molecules such as mRNAs, lncRNAs, pseudogenes, circular RNAs are included. In miRsponge, we manually curate miRNA-sponge and ceRNA interaction data through high confidence experiments across 11 species. We found that there were only 2, 5 and 10 associations in miRSponge in comparison with starBase, DIANA-LncBase and LncACTdb, respectively. In addition to miRNA sponges, lncRNAs can also sequester miRNAs by harboring the miRNA locus. For example, H19 and its mature product miR-675 are co-expressed, and regulate gastric cancer development through the recently discovered H19/miR-675/RUNX1 pathway (41). These associations were also identified using miRSponge, confirming the usefulness of the database. In combination with potent and versatile vector technologies, custom-designed sponges are already established as proficient therapeutic gene vessels in the clinic (19). In biological and medical analysis, miRNA target interactions identified from experimental validation and computational methods are both important resource. These databases play complementary roles to each other. Notably, ceRNAs are not always functionally consequential (42, 43). Denzler *et al*. (42) have quantitatively released target repression in a threshold-like manner at high target site abundance, and found the threshold was insensitive to the effective levels of miR-122 in primary hepatocytes and livers. Deepening study and development of advanced experimental results will provide worthwhile information and perspective of ceRNAs.

In summary, the miRSponge database provides a timely and valuable resource that can significantly improve our understanding of transcriptome communication among different RNA classes, and will aid future miRNA research and clinical applications.

## Funding

This work was supported in part by the National High Technology Research and Development Program of China [863 Program, 2014AA021102], the National Program on Key Basic Research Project [973 Program, 2014CB910504], the National Natural Science Foundation of China [91439117, 61473106 and 31401090], the Postdoctoral Science Foundation of China [2015M571432] and Postdoctoral Foundation of Heilongjiang Province [LBH-Z14148]. Funding for open access charge: National High Technology Research and Development Program of China [863 Program, 2014AA021102], the National Program on Key Basic Research Project [973 Program, 2014CB910504], the National Natural Science Foundation of China [91439117, 61473106 and 31401090].

*Conflict of interest*. None declared.
